# Metformin and temozolomide, a synergic option to overcome resistance in glioblastoma multiforme models

**DOI:** 10.18632/oncotarget.23028

**Published:** 2017-12-06

**Authors:** Silvia Valtorta, Alessia Lo Dico, Isabella Raccagni, Daniela Gaglio, Sara Belloli, Letterio S Politi, Cristina Martelli, Cecilia Diceglie, Marcella Bonanomi, Giulia Ercoli, Valentina Vaira, Luisa Ottobrini, Rosa Maria Moresco

**Affiliations:** ^1^ Tecnomed Foundation and Medicine and Surgery Department, University of Milan-Bicocca, Monza, Italy; ^2^ Institute of Molecular Bioimaging and Physiology (IBFM), CNR, Segrate, Italy; ^3^ Experimental Imaging Center, IRCCS San Raffaele Scientific Institute, Milan, Italy; ^4^ SYSBIO.IT, Centre of Systems Biology, Milan, Italy; ^5^ Department of Pathophysiology and Transplantation (DEPT), University of Milan, Milan, Italy; ^6^ Imaging Core, IRCCS San Raffaele Scientific Institute, Milan, Italy; ^7^ University of Massachusetts Medical School, Worcester, MA, USA; ^8^ Hematology/Oncology Division and Radiology Department, Boston Children's Hospital, Boston, MA, USA; ^9^ Division of Pathology, Fondazione IRCCS Ca’ Granda Ospedale Maggiore Policlinico, Milan, Italy

**Keywords:** glioblastoma multiforme, metformin, temozolomide, glioma stem cells, metabolism

## Abstract

Glioblastoma multiforme (GBM) is the most aggressive primary brain tumor with poor survival. Cytoreduction in association with radiotherapy and temozolomide (TMZ) is the standard therapy, but response is heterogeneous and life expectancy is limited. The combined use of chemotherapeutic agents with drugs targeting cell metabolism is becoming an interesting therapeutic option for cancer treatment. Here, we found that metformin (MET) enhances TMZ effect on TMZ-sensitive cell line (U251) and overcomes TMZ-resistance in T98G GBM cell line. In particular, combined-treatment modulated apoptosis by increasing Bax/Bcl-2 ratio, and reduced Reactive Oxygen Species (ROS) production. We also observed that MET associated with TMZ was able to reduce the expression of glioma stem cells (GSC) marker CD90 particularly in T98G cells but not that of CD133. *In vivo* experiments showed that combined treatment with TMZ and MET significantly slowed down growth of TMZ-resistant tumors but did not affect overall survival of TMZ-sensitive tumor bearing mice. In conclusion, our results showed that metformin is able to enhance TMZ effect in TMZ-resistant cell line suggesting its potential use in TMZ refractory GBM patients. However, the lack of effect on a GBM malignancy marker like CD133 requires further evaluation since it might influence response duration.

## INTRODUCTION

Temozolomide (TMZ), used as standard therapy for glioblastoma multiforme (GBM) [1], is an alkylating agent that exerts its antitumor action through the methylation of DNA. TMZ is particularly effective against GBM tumors lacking the expression of DNA repair enzyme, O6-methylguanine-DNA methyltransferase (MGMT), which antagonizes the effect of alkylating agents. However, also MGMT-methylated tumors have been shown to finally become TMZ-resistant [2–4] and despite some initial response, GBM prognosis is poor and novel therapeutic strategies are largely needed. A number of targeted therapies administered alone or in combination is under evaluation. However, most of these drugs act on pathways that are heterogeneously expressed in tumors and this limits their efficacy [5]. A common hallmark of GBM is represented by an aberrant metabolic phenotype characterized by increased glucose demand and aerobic glycolysis (the so called Warburg effect) [6]. According to recent evidences, high glucose energy demand favors glioma stem cells (GSC) survival, through the expression of high affinity glucose transporter 3 (GLUT3) in these cells [7]. Indeed, enhanced glycolysis and over expression of glucose transporters, particularly GLUT3 and hexokinase II represent negative prognostic factors for patients with GBM [7, 8]. For this reason, targeting of cell metabolism represents an attractive therapeutic strategy for GBM [9].

Adenosine Monophosphate-Activated Protein Kinase (AMPK) is a key regulator of cell energy status that influences cell growth, proliferation, protein and fatty acid synthesis and malignant transformation [10]. AMPK is activated by alkylating agents including TMZ and its activation involves O6-methylguanine production [11, 12]. Metformin (MET), an AMPK modulator, is a biguanide commonly used as first line therapy for type II diabetes. The antineoplastic effect of MET has been recently evaluated in experimental subcutaneous model of GBM and in gliospheres showing a synergistic activity with TMZ [13, 14].

Molecular mechanism of MET is heterogeneous and not fully understood. MET affects metabolism either indirectly, acting on systemic levels of insulin or glucose, or directly, targeting energy related pathways. Indeed, it has been observed that MET reduces several factors that favor GBM progression such as cell metabolism, mitochondrial oxygen consumption and expression of Hypoxia Inducible Factor 1α (HIF-1α) [15]. In tumors, MET affects cell growth and survival, in particular blocking cell cycle progression in G0/G1, promoting cell death and inhibiting angiogenesis and tumor diffusion [16]. As previously stated, MET action is mainly associated with a direct or indirect activation of AMPK that leads to the down regulation of mTOR complex 1 (mTORC1). MET can also act on p53 and down regulate mTORC1 with AMPK independent mechanisms involving the amino acid sensor Rag GTPase and REDD1, a hypoxia dependent mTORC1 inhibitor [17]. Moreover, MET influences cell energy also blocking the mitochondrial complex I with a mechanism which differs from that of rotenone as indicated by the inhibitory effect on ROS generation [18]. The control of the GSC population is considered a key challenge in GBM [19, 20]. MET given alone or in combination with TMZ has shown a preferential inhibition of GSC also in experimental GBM models [14, 21–23]. It has been reported that *in vivo* administration of biguanides inhibits orthotopic tumor formation, by blocking stem-like glioma-initiating cells [22, 24].

Despite these promising results, antitumor effects of MET, particularly in TMZ-resistant glioma cells remain poorly documented. In this study, we investigated both the *in vitro* and *in vivo* effect of MET given alone or in combined treatment in the TMZ-sensitive U251 and in the TMZ-resistant T98G glioma cell lines. In particular, we focused our experimental investigation on the effects of MET on stem cells related markers, metabolism and apoptosis.

## RESULTS

### *In vitro* MET treatment improved sensitivity of U251 and overcame T98G resistance to TMZ

**Figure 1:**
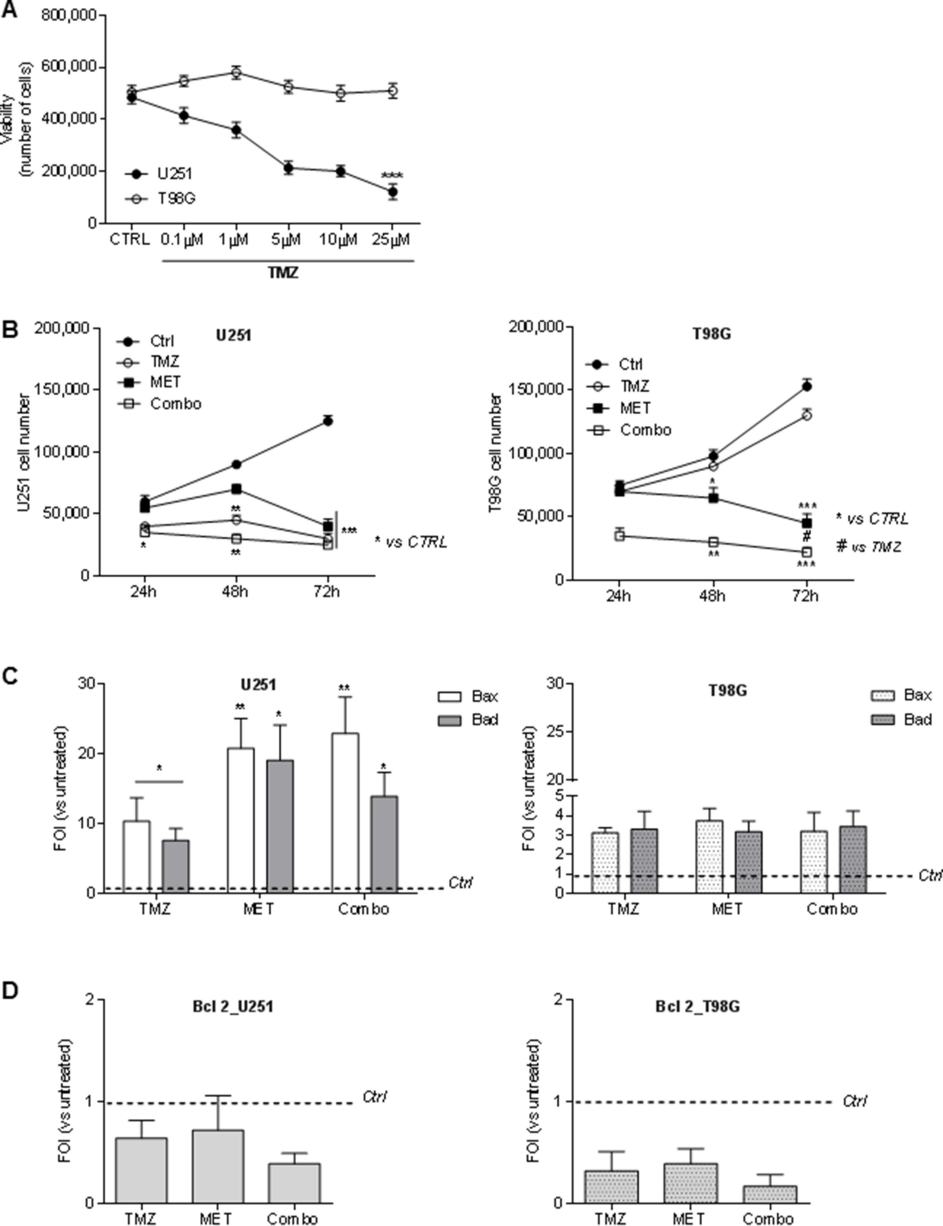
Temozolomide and metformin effects on U251 and T98G cells viability and apoptosis (**A**) U251 and T98G cells were treated with increasing doses of TMZ (0–25 μM) for 48 hours. Cell viability was assessed by Trypan blue exclusion test and expressed as number of cells. (**B**) MET for 24, 48 and 72 h. Cell viability was assessed as previously described.U251 and T98G cells were treated with 25 μM TMZ and/or 10 mM MET for 24, 48 and 72 h. Cell viability was assessed as previously described. (**C**) Real time-PCR for pro-apoptotic *Bax* and *Bad* genes 48 h after treatment with TMZ and/or MET. Data were normalized for β-actin and ΔΔct expressed as Fold Of Induction (FOI). (**D**) Real time-PCR for anti-apoptotic *Bcl-2* gene 48 h after treatment with TMZ and/or MET. Data were normalized for β-actin and ΔΔct expressed as Fold Of Induction (FOI). Data are shown are mean ± standard deviation. ^*^p < 0.05; ^**^ *p* < 0.01; ^***^p < 0.001 *vs* control sample (untreated cells). ^#^*p* < 0.05 *vs* TMZ treatment.

In order to determine the dose of TMZ able to discriminate U251 and T98G responsiveness, both cell lines were firstly exposed to different doses of TMZ for 48 h(Supplementary Figure 1). and cell viability was determined by Trypan blue exclusion test. A TMZ dose of 25 μM was defined as the optimal dose to distinguish drug sensitivity of the two cell lines (Figure 1A). Furthermore, to determine whether MET affects tumor cell proliferation or increases sensitivity to TMZ, U251 and T98G cells were treated for 24, 48 and 72h with MET, TMZ and their combination (Combo). U251 cells showed a time-dependent progressive inhibition of cell growth that was particularly evident with TMZ or Combo treatment but also visible with MET at 72h (Figure 1B, left panel). As expected, T98G cells were not sensitive to TMZ. However, both MET and Combo significantly reduced cell proliferation (Figure 1B, right panel). While in U251 cells, the addition of MET to TMZ determined only an additive effect, in T98G cells MET synergistically acted with TMZ, triggering inhibition levels ranging from 34% (MET only) to 69% (Combo) at 48 h (Supplementary Figure 1).

### MET or combo treatment increased Bax and Bad transcripts but not Bcl-2

In order to assess the effect of the drugs on cell apoptosis, pro-apoptotic *Bax* and *Bad* and anti-apoptotic *Bcl-2* genes were analyzed using Real time-PCR. In U251 cells, TMZ treatment induced an increase in Bax and Bad transcripts that were significantly higher after the administration of MET or Combo treatment (Figure 1C). Furthermore, Annexin V levels detected by FACS analysis were higher in Combo treated cells compared to untreated cells (75 ± 24 vs 10.4 ± 4.4). In T98G TMZ resistant cell line, effect of MET or Combo was similar to that one observed after TMZ treatment for both *Bax* and *Bad* transcripts (Figure 1C). However, COMBO treated cells showed an increase of Annexin V levels compared to control cells (35 ± 12 vs 13.3 ± 6.1). In both cases, no significant modulation of activated/cleaved Caspase-3 was observed (data not shown).

Finally, in both cell lines we did not observe a significant variation in *Bcl-2* expression in all the drug regimens considered herein (Figure 1D). However, after Combo treatment, a deeper increase of *Bax/Bcl-2* and *Bad/Bcl-2* ratio, in both cell lines compared to single treatments was observed (Table 1).

**Table 1:** Bax and Bad to Bcl-2 ratios in U251 and T98G cell line

Cell line	Treatment	Bax/Bcl-2	Bad/Bcl-2	*P* value (vs control)
U251	TMZ	16.09 ± 2.5	11.8 ± 3.6	0.05
	MET	28.8 ± 3.6	26.4 ± 2.6	0.05
	TMZ + MET	58.6 ± 2.5	35.6 ± 2.5	0.001
T98G	TMZ	9 ± 1.5	10.0 ± 2.2	0.05
	MET	9 ± 2.2	8.0 ± 1.8	0.05
	TMZ+MET	18 ± 3.0	20.0 ± 2.6	0.05

TMZ + MET vs single treatments *p* < 0.001

Data are expressed as mean ± SD.

### MET and combo treatment differently modulated GBM markers exerting a preferential effect on CD90

To evaluate if MET could act on a specific population of GBM cells, we measured the effect of TMZ, MET or Combo on the relative abundance of two markers: CD133 and CD90. The former, although not selectively expressed in cancer related stem cells, contributes to treatment resistance and tumor formation; the latter is widely expressed by a variety of cancer stem cells and according to some authors also in GSC [25, 26].

**Figure 2:**
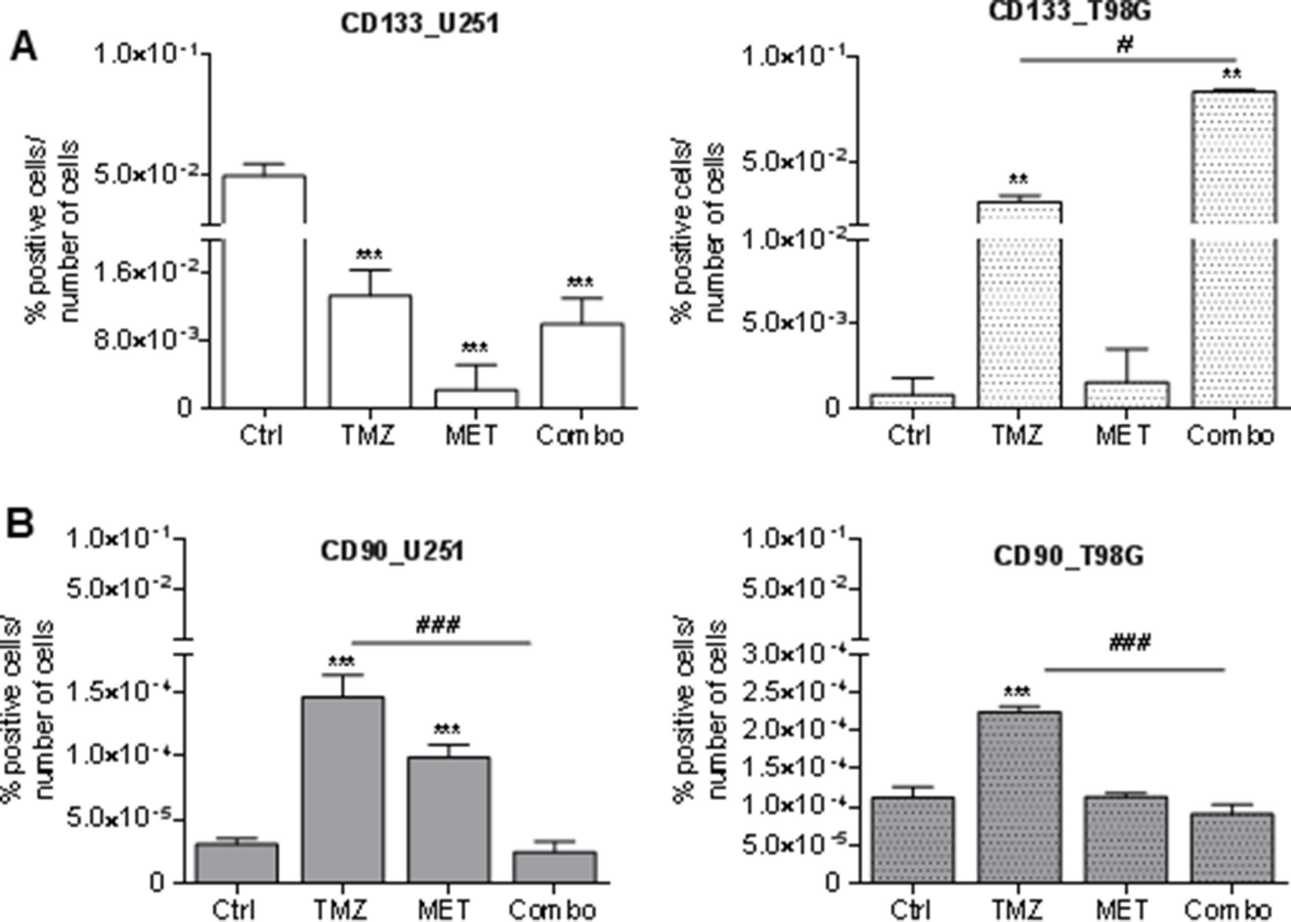
Glioma stem cell marker modulation after treatments FACS analysis for CD133 (**A**) and CD90 (**B**) markers in U251 and T98G cells after 48 h of treatment with 25 μM TMZ and/or of MET. Data were expressed as percentage of positive cells on the number of total cells. Data are shown are mean ± standard deviation. ^**^p < 0.01; ^***^p < 0.001 *vs* control sample (untreated cells). ^#^*p* < 0.05; ^###^*p* < 0.001 *vs* TMZ treatment.

A high percentage of U251 cells were CD133 positive at baseline. Administration of MET significantly reduced CD133 positivity of U251 cells and this effect was higher than that one observed after TMZ or Combo treatment (Figure 2A). In T98G cells, we observed a low level of CD133 in untreated condition, but a TMZ dependent increase in CD133 that was even higher after MET co-administration. No effect was observed in MET treated cells. TMZ significantly increased CD90 expressing cells in both cell lines but co-administration of MET reverted this effect (Figure 2B). When given alone, MET increased CD90 only in U251 cells. Similar data were also obtained using Real time-PCR supporting the increase of CD133 and CD90 mRNA transcription (Supplementary Figure 2A–2D).

To further understand if combined treatment could affect other glioma subpopulations markers, FACS analysis of CD44 and CD73 were performed on both U251 and T98G cells (Supplementary Figure 3). CD73 modulates cell adhesion, migration and invasion [27] and contributes to local adenosinergic immune suppression by acting on infiltrating CD4^+^T lymphocytes [28]. CD44 is mainly expressed by the mesenchymal subtype of GBM cells and seems to exert a crucial role in tumor initiation and progression [29, 30]. TMZ, but not MET, reduced the relative expression of CD73 only in U251 cells (Supplementary Figure 3A and 3B). In addition, TMZ increased the relative abundance of CD44 in both cell lines, an effect that was not counteracted by drugs co-administration (Supplementary Figures 3C and 3D). In summary, MET reduced the relative expression of CD90 and CD133, the latter only in U251, but it was not able to counteract for the selection of CD133 and CD44 induced by TMZ.

Finally, we also investigated Sox2, an oncogene which plays important roles in cancer stem cells (CSC) and in TMZ-resistance in glioma [31]. In U251-sensitive cell line, Real time-PCR analysis showed a significant decrease in Sox2 expression after COMBO treatment, 8.8-fold, (*p* < 0.01), when compared to TMZ and control treatment. Moreover, in T98G-resistant cell line, we observed a huge increase in Sox2 expression after TMZ treatment (18-fold compared to control, *p* < 0.0002) that was significantly counteracted of 3.5-fold (*p* < 0.0001) by Combo treatment.

### MET treatment reduced ROS levels but promoted the glycolytic phenotype of GBM cells

**Figure 3:**
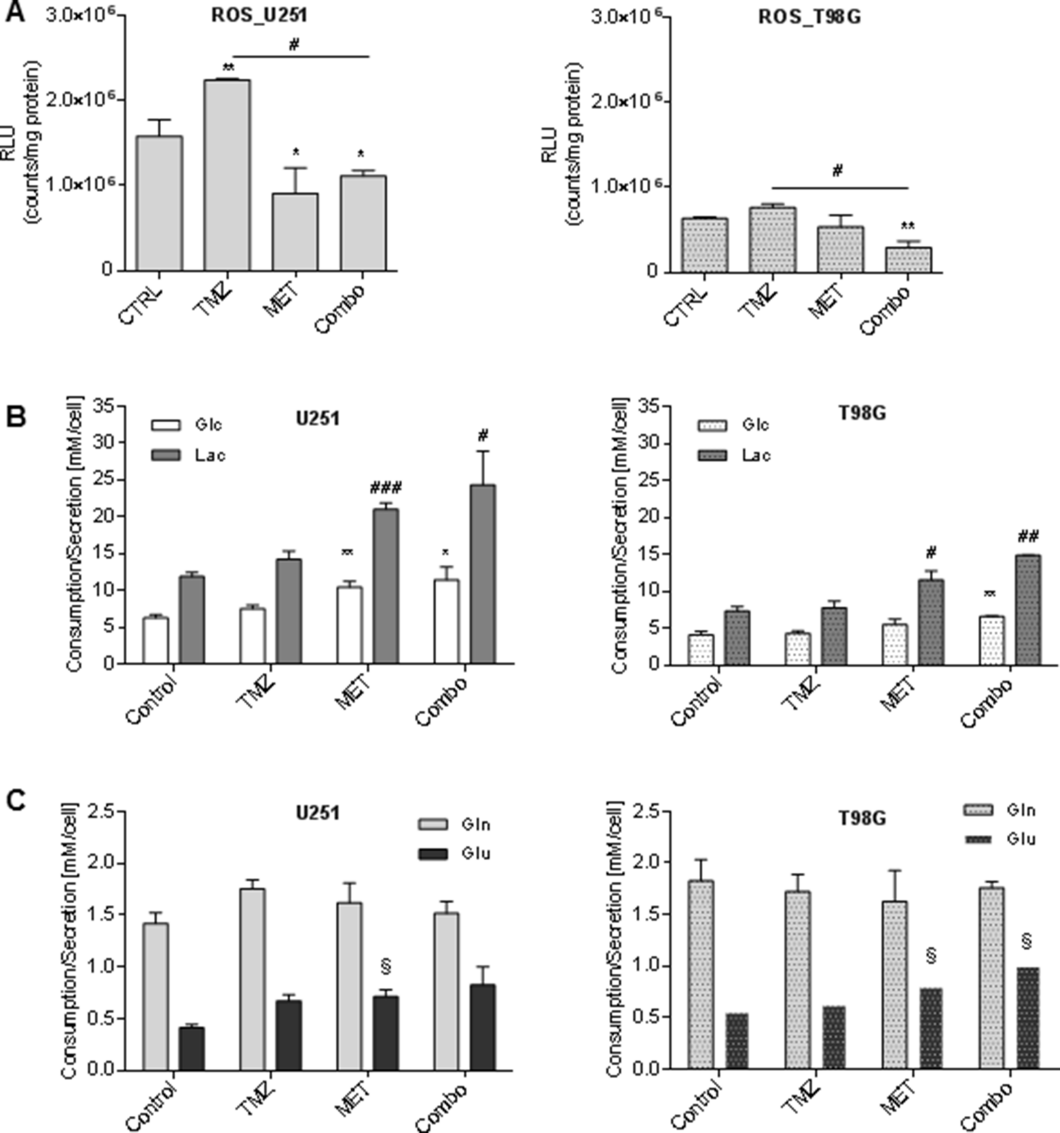
Metformin modulated ROS production and glucose and glutamine metabolism (**A**) Luminescent assay applied to measure the level of hydrogen peroxide (H_2_O_2_) in cell culture medium of U251 and T98G after treatment with 25 μM TMZ and/or 10 mM MET. Data were expressed as Relative Luminescence Units (RLU) obtained by luciferase counts normalized to amount of proteins quantified by Bradford assay. (**B**) Glucose lactate, (**C**) glutamine and glutamate were measured in cell medium after 48 h of therapy. Data are shown as mean ± standard deviation. ^*^p < 0.05; ^**^p < 0.01;^***^p < 0.001 *vs* control sample (untreated cells); ^#^*p* < 0.05; ^##^*p* < 0.01; ^###^*p* < 0.001 *vs* control sample; ^§^ *p* < 0.05 vs control sample (untreated cells).

Evidences in literature indicate that MET inhibits mitochondrial complex I but it does not increase reactive oxygen species (ROS) levels [15]. Here we confirmed this observation reporting a MET-dependent reduction of ROS levels and an impairment of TMZ-dependent up-regulation of ROS in both cell lines (Figure 3A).

To evaluate how TMZ, MET or Combo affected cell metabolism, we measured glucose and glutamine consumption related to lactate and glutamate secretion. Neither of cell lines showed significant metabolic changes due to TMZ alone administration. On the contrary, MET administration, alone or in association with TMZ, was accompanied by an increase in the rate of glucose uptake and lactate secretion in U251 cancer cells (Figure 3B left panel) as compared to T98G cells (Figure 3B right panel). Furthermore, we did not observe a significant effect of the drugs in glutamine uptake but we detected a slight increase of glutamate secretion in both cell lines associated with MET administration (Figure 3C).

### Repeated *in vivo* administration of MET increased the sensitivity of T98G model to TMZ

**Figure 4:**
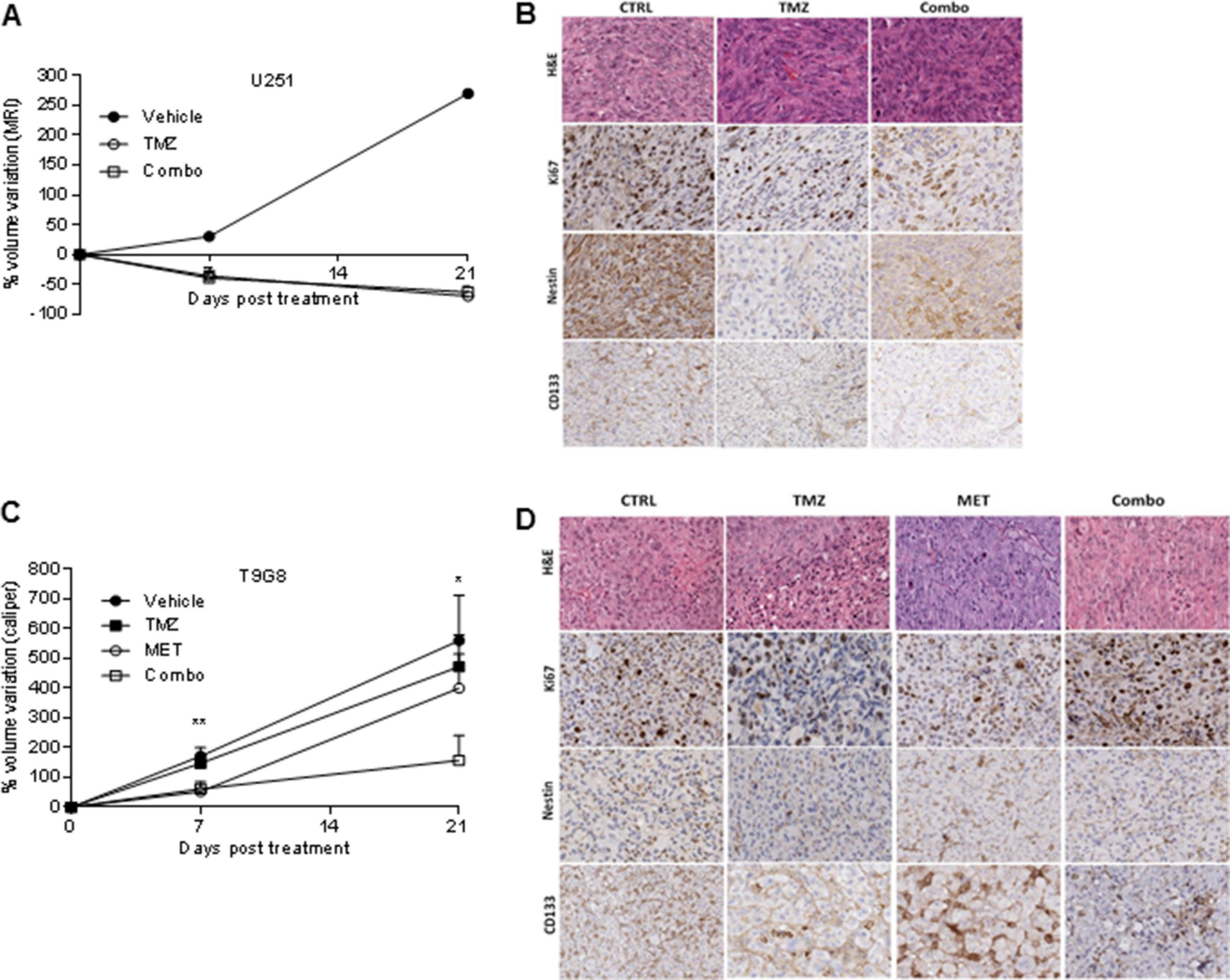
MET and TMZ effects in *in vivo* glioma models (**A**) Variation of U251 tumor volume measured at contrast-enhanced T1 MRI after treatment expressed as percentage of volume change. Error bars indicate SEM. (**B**) U251 derived orthotopic brain tumors from vehicle (CTRL), TMZ or TMZ/metformin (Combo) treated mice were analyzed for Ki67, Nestin and CD133 markers by immunohistochemistry. No difference between the tumor edge and bulk areas in IHC staining intensities or percentages of positive cells was observed for any of the investigated markers. H&E was performed for morphological evaluation of the tumors. Representative images are shown with original magnification x200. (**C**) Variation of T98G tumor volume measured using caliper after treatment expressed as percentage of volume variation. In T98G xenograft model already at 7 days, the combined treatment significantly slowed down tumor growth compared to control (*p* value = 0.005), moreover a transient reduction was observed with MET (*p* value = 0.03). After 3 weeks, only Combo-treated mice displayed tumor smaller than vehicle (*p* value = 0.04). Error bars indicate SEM. (**D**) T98G tumors from vehicle (CTRL), TMZ, MET or TMZ/metformin (Combo) treated mice, were analyzed for Ki67, Nestin and CD133 markers expression by immunohistochemistry. No difference between the tumor edge and bulk areas in IHC staining intensities or percentages of positive cells was observed for any of the investigated markers. Hematoxylin and eosin staining (H&E) was performed for morphological evaluation of the tumors. Representative images are shown with Original magnification x200.

To test if MET was able to revert resistance of T98G cells to TMZ and to increase the response of U251 cells when administered *in vivo*, we compared the efficacy of TMZ administered alone to that of TMZ in combination with 250 mg/kg i.p. MET in both GBM models. This dose of MET was selected on the basis of previous reports [32] and on the lethal toxicity observed with higher doses. In general, TMZ and MET association was well tolerated. In the first days of therapy, TMZ induced a transient reduction of mouse weight, which was exacerbated by the addition of MET, but weight was rapidly recovered in the following days. U251 vehicle mice lost their weight due to tumor progression (Supplementary Figure 4B and 4C). TMZ alone or Combo were administered in mice bearing intracranial U251 GBM cells and their effects were monitored *in vivo* using MRI. A progressive reduction in tumor volume growth was observed in mice treated with TMZ independently from MET (Figure 4A). TMZ in combination and TMZ alone triggered a comparable reduction of tumor volume (-39.0 ± 29.3% and -35.9 ± 24.6% respectively) compared to vehicle (+30.4%) as depicted by MRI analyses performed 7 days after treatment (Figure 4A and Supplementary Figure 4A). Both treatment regimens displayed a similar efficacy at day 21: -62.9 ± 27.3% and -69.5 ± 17.8% in Combo and TMZ, respectively. At 41 days, a marked increase in tumor volume was observed in the majority of mice, except for two animals treated with Combo showing an arrest of tumor growth at MRI. In an additional group of mice treated with MET alone, we did not observe any significant difference compared to mice treated with vehicle (data not showed). Ex vivo IHC analyses showed a significant decrease in CD133 marker in Combo group compared to untreated group (*p* = 0.028), confirming *in vitro* results (Figure 2A and Figure 4B), but no difference was observed compared to TMZ treated group. However, no significant difference between TMZ and Combo groups in the expression of Ki67 and Nestin was observed (Figure 4B and Supplementary Figure 5A).

As expected, in T98G tumor bearing mice, TMZ treatment was not able to reduce tumor growth. On the contrary, we observed a synergistic effect of TMZ in association with MET as indicated by the significant reduction in tumor growth rate in Combo group (TGI increased from 11% to 52%). The group of mice treated with MET showed a transient reduction in the rate of tumor volume increase similar to that observed for Combo animals, but only during the first week of treatment (Figure 4C).

In IHC performed on samples obtained at 21 days, we observed a significant increase in Ki67 marker in Combo group compared to vehicle probably anticipating a relapse of tumor growth (*p* = 0.0043) (Figure 4D and Supplementary Figure 5B). Nestin and CD133 were similarly expressed in control and Combo groups.

**Figure 5:**
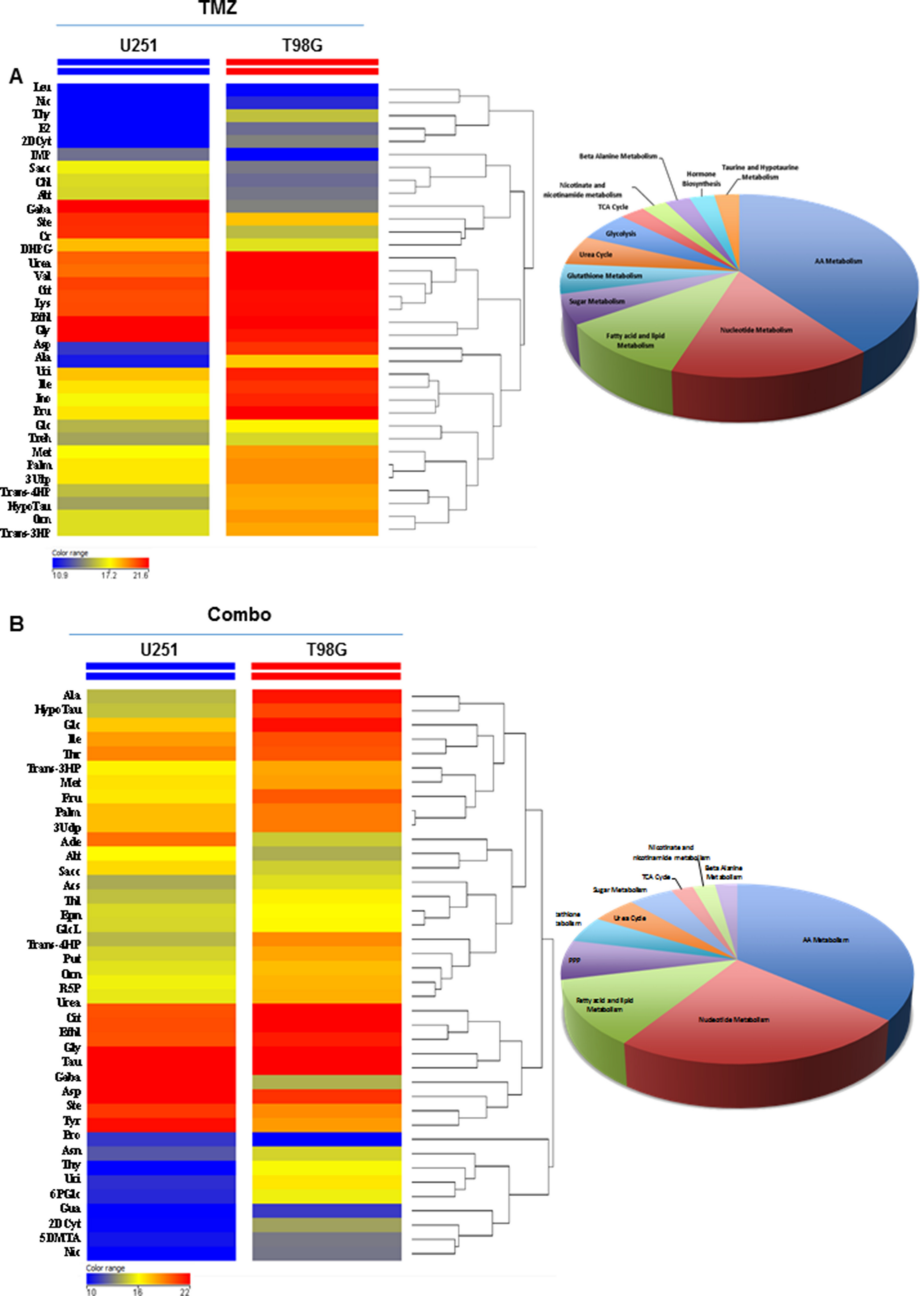
Comparison of drug metabolic effects in *in vivo* glioma models (**A**) Metabolic profiling comparison between *in vivo* U251 and T98G samples treated with TMZ by *t*-test statistical analysis. (**B**) Metabolic profiling comparison of *in vivo* U251 and T98G samples treated with TMZ plus MET by *t*-test statistical analysis. *T*-test statistical analysis was performed using MPP software. The dendrogram was produced by applying a hierarchical clustering algorithm. The legends color range was automatically generated by MPP, considering the minimum and maximum values of most compounds identified to highlight the best differences between samples through the most suitable color scale.

To further investigate the impact of treatment on *in vivo* metabolism remodeling, we performed metabolic profile of tumors using gas-chromatography-mass spectrometry technique. Metabolomics statistical analysis was performed using a mass profiler professional *t*-test and the result of the analysis represented significant metabolites in hierarchical clustering showing similar abundance values considering the minimum (blue color) and maximum (red color) color scale within a tree structure. The untargeted post mortem metabolic profiling of U251 mouse model showed, in treated animals, a notable metabolites set enrichment related to amino acids, fatty acids and lipids metabolism. In addition, analysis displayed a significant reduction of 32 metabolites in Combo when compared to TMZ alone (Supplementary Figure 6A). The metabolic profiling performed post mortem in T98G tumor mouse model identified significant differences between TMZ alone and Combo in 8 compounds belonging to nucleotides, amino acids, glutathione, lipids, amino sugar and β-alanine metabolism (Supplementary Figure 6B). Finally, to confirm the different effect of drugs in the two mouse models, we compared the metabolic profile of the two groups of animals including treatment effects. The untargeted metabolic profiling indicated a significant reduction of metabolites in U251 mice in comparison with T98G independently from treatment, and this reduction concerned metabolites belonging to nucleotide and amino acids pathway (Figure 5A–5B).

## DISCUSSION

In the present work, we investigated the effect of metformin, a first line antidiabetic drug with promising anticancer effects on GBM models, when given alone or in association with TMZ-based standard chemotherapy. To this aim, treatment effects were evaluated *in vitro* and *in vivo* in two GBM models characterized by different MGMT methylation status and TMZ sensitivity. Recent evidences suggest that MET is able to i) modulate anabolic metabolism, inducing cell cycle arrest and cell death [13, 33]; ii) reduce GSC cells, increasing the treatment sensitivity [22] and iii) improve responsiveness of glioma cells to TMZ treatment, counteracting the MGMT presence [13]. The efficacy of combining MET and the alkylating agent TMZ in GBM has been already proposed [34] since it increases AMPK activation. In addition, according to published data, combined treatment with TMZ and MET exerts a greater inhibition on glioma cancer stem cells compared to single agents [14, 21]. Based on these observations, we further investigated *in vitro* and *in vivo* the effect of MET given alone or in combination in the TMZ-sensitive U251 GBM cells and explored its potential efficacy on the TMZ-resistant T98G GBM cells. *In vitro* we showed that MET reduced growth rate of both U251 and T98G cells with an additive effect for the former and a synergistic one for the latter when given in association with TMZ.

Both TMZ and MET modulated pro- and anti-apoptotic genes without affecting Caspase-3 activation. Although a clear mechanism for MET-induced apoptosis has not been clarified, Zhuang et colleagues have already demonstrated that in MET-sensitive cancer cell lines, cell death was mediated by both caspase-dependent and -independent mechanisms, these last involving poly(ADP-ribose) polymerase (PARP) [35]. Our results showed that the increase in the regulation of pro-apoptotic genes, detected especially in TMZ-sensitive cells, was not caspase-dependent, since the levels of cleaved Caspase-3 were not affected by treatments (data not shown). In parallel, we observed a down-regulation of Bcl-2 associated to an increase of Bax resulting in a significant increase of Bax/Bcl-2 ratio.

Oxidative stress triggered by chemotherapeutic agents, as TMZ, is considered a putative mediator of apoptosis [36]. We observed an increase of ROS production after TMZ treatment (Figure 3A) but only in U251 cell line. On the contrary, the addition of MET to TMZ leads to a decrease of ROS both in TMZ-sensitive and in -resistant cell line. It has been previously observed that MET inhibits mitochondrial ROS production, a mechanism that has been related to the blockage of the reverse electron flow through the respiratory chain complex I [18, 37, 38]. Dysfunction of the respiratory chain complex I could exacerbate metabolic need and Warburg effect. Regarding this effect, we found that the use of MET alone and in combination with TMZ also induced significant changes in glucose metabolism in both cell lines. In particular, it prompted a higher consumption of glucose and a higher release of lactate in cell culture (Figure 3B) indicating that MET exacerbates Warburg effect as previously observed [13, 23].

This metabolic reprogramming is an index that cells are more susceptible to the lack of energy supply. Glutamine could represent an alternative energetic substrate to fuel cancer cells. After MET administration, we observed a slight increase of glutamate levels in extracellular medium of T98G cells but no modification in glutamine uptake. This last observation is not surprising since GBM can produce glutamine from intracellular glutamate through the activity of glutamine synthetase and glutamate is released outside cells also during glutamine starvation [39]. Glutamate release occurs via X^c^ system, a cysteine-glutamate exchanger that imports cysteine for the synthesis of the cellular antioxidant glutathione (GSH) and protects cells from cytotoxic intracellular glutamate levels [40, 41]. On the other hand, it is well known that a significant increase in the quantity of glutamate released in the extracellular space contributes to excitotoxicity and consequent glioma expansion in the peri-tumoral space [40]. The increased outflow observed in T98G cells may occur to prevent a potential glutamate overload due to the reduction of anabolic processes induced by MET.

A large number of evidences showed that MET affects GSC proliferation, self-renewal capabilities and survival with a higher efficiency compared to differentiated glioma cells [14, 23]. In the present study, we focused our attention on different putative negative prognostic markers of GBM linked with GSC or GIC (glioma initiating cells): CD133, CD90 and CD44. CD133 is largely used as a negative prognostic CSC marker in several tumors, including GBM [42], although its function in normal and cancer stem cell is not definitely understood. CD90, also known as Thy-1, is a heavily glycosylated glycophosphatidylinositol (GPI)-anchored cell surface protein that has been previously identified as a marker for several stem cells such as hematopoietic stem cells and bone marrow-derived mesenchymal stem cells. Nevertheless, its role in GSC is still under debate [43]. In any case, the expression of CD90 dramatically raises in case of tumor shift from low to III-IV grade glioma considering it a marker of high-grade brain malignancy. On the other hand, CD44 seems to exert a crucial role in tumor initiation and progression. Our *in vitro* results indicated that MET alone strongly decreased (in U251) or maintained stable (in T98G) the relative abundance of CD133 cells but not in Combo treatment where the effect of TMZ became prevalent. A similar result was observed also for the CD44 marker. On the contrary, MET was able to significantly counteract TMZ effect, decreasing the relative abundance of CD90 cells in Combo group both of U251 and T98G cells, suggesting a role of MET in the modulation of this population of cells independently from TMZ-sensitivity.

In our *in vivo* experiments, U251 cells confirmed their high sensitivity to TMZ, indeed TMZ alone was able to significantly reduce tumor volume until 21 days from the beginning of therapy similarly to Combo treatment (Figure 4A). In U251 the effect of MET could be partially covered by the high dose of TMZ (400 mg/kg) which we have chosen to compensate the absence of radiotherapy. Conversely, in T98G tumors TMZ alone didn't cause any effect although the association of TMZ and MET was able to synergistically reduce the rate of tumor growth compared to control. Nevertheless, at day 21 a relapse of tumor growth was visible in Combo tumors. In addition, also MET alone decreased tumor growth in the first 7 days of therapy. Metabolic analysis performed on tumor samples collected post mortem confirmed therapy modulation of tumor metabolism. Main effects were due to TMZ, indeed metabolomics analysis showed significant changes in amino acid, nucleotide and fatty acid and lipid metabolism. In addition a higher response both to TMZ alone and to Combo was observed in U251 tumors than in T98G ones (Figure 5A–5B) indicating a higher sensitivity of U251 to this therapy and a recover of growth of T98G tumors at 21 days. Stem cells markers could explain the lack of long-term effects exerted by the combined administration of the two drugs. MET showed a high effect on CD90 expressing cells, but it was not able to contrast the increase in the relative abundance of CD133 and CD44 induced by TMZ. The time-dependent selection of these aggressive phenotypes could limit the duration of treatment efficacy even in presence of MET. On the other hand, other factors may affect the *in vivo* response. The human Organic Cation Transporters (OCT1, OCT2, OCT3), which are expressed on cell membrane, influence MET uptake in different tissue compartments including tumor. OCTs are synthesized in the cytoplasmic region and during time, due to the constant intracellular trafficking, are partially moved and inserted to the external membrane. A time-dependent reduction or miss-localization of OCT3 has been reported in glioma cells orthotopically implanted in mice [44]. For these reasons, an involvement of the OCT family in the duration of MET effects cannot be excluded. In addition, tumor-associated inflammatory response is pivotal to support growth and invasion and could have an important role in chemoresistance [45]. New PET radiopharmaceuticals have been developed to detect the 18 kDa translocator protein TSPO which is expressed by most glioma cells and activated microglia [46]. Nevertheless, our athymic models limit the possibility to fully investigate the complex interaction between inflammation cells and glioma cells by underling the need to perform further studies in syngeneic models.

In conclusion, results of our study showed MET efficacy in reverting treatment resistance in a TMZ-insensitive cell line as indicated by the *in vitro* and *in vivo* results on T98G cells model suggesting the potential usefulness of MET for the treatment of TMZ refractory GBM patients. In U251-TMZ sensitive cells we observed that MET alone or co-administered with TMZ is more effective than TMZ alone *in vitro*. However, when administered *in vivo*, differently to what previously showed in patients-derived GIC [22], MET was not able to block tumor growth or to potentiate the effects of a high dose of TMZ. Our results indicate that the *in vivo* effect of MET is cell line dependent and it may be transient. These observations stress the need for a further experimental development of MET in a large set of well-characterized GBM models.

## MATERIALS AND METHODS

### Cell lines and reagents

The human U251 (kindly provided by Dr. G. Melillo, National Cancer Institute, Frederick, MD, USA) and T98G (ATCC, Manassas, VA, USA) glioblastoma cell lines were transfected to put the expression of mCherry protein under the control of PGK promoter. U251 and T98G cells were routinely maintained in RPMI or E-MEM medium supplemented with 10% heat-inactivated fetal bovine serum, penicillin and streptomycin (50 IU/ml) and 2mM glutamine (Euroclone, UK). Cells were maintained in a humidified atmosphere of 5% of CO_2_ at 37°C. U251 and T98G cells displayed a methylation of MGMT promoter of 65% and 40%, respectively measured using pyrosequencing analysis. *In vitro* treatments were performed as follows: 10,000 cells/cm^2^ were treated with different concentrations of TMZ (0.1, 1, 5, 10, 25 μM) and 10 mM of MET (both Sigma Aldrich, St. Louis, MO, USA) for 24, 48 and 72 hours (h). Cell viability was evaluated by Trypan blue exclusion test. The effect of MET and TMZ in combination was calculated considering cell growth inhibition measured as: [1 - (C_f_ / C_0_)_A_ / (C_f_ / C_0_)_V_)]^*^ 100 where C_f_ is the cell number at the point analyzed, C_0_ is the cell number at the beginning of treatment, A is the corresponding drug and V is the vehicle [47].

### FACS analysis

Two hundred thousand (200,000) cells were washed in PBS and incubated with 0.5 μg of: CD90-FITC, human (clone: DG3)-FITC, human 130-095-403; Monoclonal CD133/2 (293C3)-FITC, human 130-090-853; CD44-FITC, mouse (clone: IM7.8.1) 130-102-511; CD73-PE, human (clone: AD2)-FITC, human 130-095-182 (MiltenyiBiotec). For apoptosis, in the same cells FITC Annexin V apoptosis Detection Kit I (BD Pharmingen) was used to measure Annexin V levels.

Viable cells were gated by forward, side scatter and analyzed on 100,000 acquired events for each sample. Samples were analyzed on a PartecCyFlow Space using the PartecFloMax^®^ software.

### RNA extraction and real-time PCR

RNA was extracted using the commercially available illustra RNAspin Mini Isolation Kit (GE Healthcare, Italy), according to manufacturer's instructions. Total RNA was reverse-transcribed to cDNA using the High Capacity cDNA Reverse Transcription Kit (Applied Biosystem, USA). Real-time PCR was performed in duplicate for each data point and the oligonucleotides used were: beta-actin (FRW: ATCAAGATCATTGCTCCTCCTGA, REV: CTGCTTGCTGATCCACATCTG); Bax (FRW: GAG AGG TCT TTT TCC GAG TGG; REV: CCT TGA GCA CCA GTT TGC TG); Bad (FRW: GTTCCAGATCCCAGAGTTTG; REV: CCTCCATGATGGCTGCTG); Bcl-2 (FRW: ′TTGTGGCCTTCTTTGAGTTCGGTG, REV: GGTGCCGGTTCAGGT ACTCAGTCA); Sox 2 (FRW: GCACATGAACGGCTGGAGCAACG; REV: TGCTGCGAGTAGGACATGCTGTAGG). Changes in the target mRNA content relative to housekeeping (β-actin) were determined with the ΔΔct method.

### Luminescence kit

U251 and T98G were treated as previously described and, after 48 h of incubation, were tested to measure the level of the ROS hydrogen peroxide (H_2_O_2_), directly in cell culture or in defined enzyme reactions. Relative luminescence units were detected using a plate reader (Glomax) and after protein normalization with Bradford assay, data were expressed as RLU (Relative Luminescence Units).

### *In vitro* evaluation of cell metabolism

To evaluate metabolic changes in response to different drugs, cell medium has been analyzed after 48 h therapies with 25 μM TMZ alone and/or 10 mM MET alone and with vehicles. Glucose, lactate, glutamine and glutamate have been measured using YSI 2950 Biochemistry analyzer (YSI, Incorporated USA). Metabolites concentration has been normalized to that of original medium and then corrected for protein concentration measured with Bradford assay.

### Mouse models

Animal experiments were carried out in compliance with institutional guidelines for the care and the use of experimental animals, which have been notified to the Italian Ministry of Health and approved by the Ethics Committee of the University of Milan and of IRCCS San Raffaele Scientific Institute of Milan. The orthotopic U251 glioma model was obtained by stereotaxic injection (coordinates: 1.5 mm lateral to the bregma, 0 mm behind, 3.0 mm ventral to the dura) [48] of 1 × 10^5^ glioma cells (U251-HRE-mCherry) in 2 μl of phosphate-buffered saline (PBS) into 7 to 8 weeks old female nude mice at day 0. To obtain the T98G glioma model, 7 to 8 weeks old female nude mice were subcutaneously (s.c.) injected on both flanks with 5 × 10^6^ T98G cells mixed 1:1 with matrigel. After cells injection, mice were monitored every day for body weight and signs of illness and sacrificed at appearance of evident signs of illness or at loss of more than 15% of the initial weight.

### Animal treatments and monitoring

As previously observed, 21 days after cells injection mice bearing U251 cells showed visible tumors and treatments began [49]. TMZ (400 mg/kg as single dose) dissolved in 10% DMSO was administered alone by oral gavage as standard therapy (TMZ group, *n* = 7) and in association with MET (i.p. 250 mg/kg, daily for 21 days) dissolved in saline (Combo group, *n* = 8). Our TMZ dose was slightly higher than that one administered in adjuvant therapy in a 60 kg patient. Indeed adjuvant TMZ therapy consists of 1620 mg/cycle for a human of average weight. According to the surface area rule, our dose consists of 1944 mg.

Tumor response was monitored using Magnetic Resonance Imaging (MRI) performed before starting treatments as well as after 7 and 21 days of treatment. After the end of therapy, mice were monitored to disease relapse and in presence of evident signs of illness, animals were sacrificed and brain removed for IHC analysis. A further group of mice was treated with vehicle (Control group, *n* = 4) and monitored for survival. For T98G model, when tumors were visible mice were randomly assigned to treatment groups (Vehicle, TMZ, MET and Combo) and tumor growth was evaluated twice a week using caliper following the formula V = (L × l^2^) / 2 where L is the longer side and l is the shorter one. Modifications in tumor volume were calculated using: TV = (V_t_-V_0_) / V_0_^*^100 (where V_0_ is the tumor volume at the beginning of treatments and V_t_ is the volume at different times). Moreover, tumor growth inhibition (TGI) and the effects of treatment combination were calculated at the last time point according to Navarro et al. [47].

### MRI study

Magnetic Resonance Imaging (MRI) was performed on a 7T preclinical magnetic resonance scanner (Bruker, BioSpec 70/30 USR, Paravision 5.1, Germany), equipped with 450 mT/m gradients (slew-rate: 3400–4500T/m/s; rise-time: 140 ms). A phased-array mouse-head coil with four phased-array channels was used as receiver, coupled with a 72 mm linear-volume coil as transmitter. Mice were anesthetized with isoflurane (2% in oxygen) and positioned prone on a dedicated heated apparatus, to prevent hypothermia. A coronal 2D High Resolution (HR) Rapid Acquisition with Relaxation Enhancement (RARE) T2 [repetition time (TR) = 3200 ms; echo time (TE) = 38 ms; rare factor = 8; field of view (FOV) = 14000 mm; matrix = 256; slice thickness/inter-slice distance = 0.6 × 0.6 mm] and a RARE T1 [TR = 438.088 ms; TE = 7.28 ms; rare factor = 2; FOV = 20000 mm; matrix = 170] were acquired. After the injection of 0.2 μl/g of gadobutrol (Gadovist, Bayer Schering Pharma, Berlin-Wedding, Germany), acquisition of the RARE T1 was repeated. Tumor volume was calculated by manual contour of the post-contrast RARE T1 sequence by a neuroradiologist with 16 years of experience in preclinical MR imaging.

### Immunohistochemistry

At sacrifice, brains were collected, fixed in 10% neutral buffered formalin (Sigma-Aldrich) and paraffin embedded. Standard haematoxylin and eosin (H&E) staining was performed for morphological evaluation.

The following specific primary antibodies were used for immunohistochemistry (IHC) experiments: HIF-1α (clone54, dilution 1:200, BD Transduction Laboratories, San Diego, CA, USA), Ki-67 (30–9 CONFIRM, dilution1:100, Ventana Medical Systems Inc., Tucson, AZ, USA), CD133 (AC133, Miltenyi Biotec, Bergisch Gladbach, Germany) and Nestin (MAB1259, dilution 1:4000, R&D Systems, Minneapolis, MN, USA). IHC was performed using an automatic stainer (BenchMark ULTRA, Ventana Medical Systems Inc.), with diaminobenzidine (DAB ultraView, Ventana Medical Systems Inc.) as chromogen, and finally slides were counter stained with haematoxylin. As negative controls, one slide per antibody was incubated only with secondary antibody and detection reagents. All slides were digitalized using Aperio Digital Pathology slide scanner (Leica Biosystems, Milan, Italy) at 20x magnification. Immunoreactivity for cytoplasmic (Nestin), membranous (CD133), or nuclear (Ki67) localization in the various samples was evaluated and scored using an automated algorithm implemented in ImageScope software (Leica Biosystems), in terms of percentages of positive cells.

### Metabolite profiling in tissue samples

Tissue metabolite extraction and derivatization were performed as described in Gaglio et al. [50]. GC/MS analysis was performed using 7200 accurate-mass Q-TOF GC/MS (Agilent Technologies) equipped with a 40-m DB-35MS capillary column operating under electron impact (EI) ionization at 70eV. Samples (1 μl) were injected in a splitless mode at 250°C, using helium as the carrier gas at a flow rate of 1 ml/min. The GC oven temperature was held at 100°C for 2 min and increased to 325°C at 10°C/min. GC/MS data processing was performed using Agilent Muss Hunter software and statistical analyses were performed using Mass Profiler Professional (MPP) software.

### Statistical analysis

*In vitro* experiments were repeated three times, giving reproducible results. Whole data are shown as mean values ± standard deviation (SD) or standard error of the mean (SEM) as indicated. *T*-test, one- or two-way analysis of variance (ANOVA) followed by Dunnett's test or Mann-Whitney U test, were performed using Prism 4 (GraphPad Software Inc., CA, USA).

## SUPPLEMENTARY MATERIALS FIGURES AND TABLES



## Abbreviations

GBM: Glioblastoma multiforme
TMZ: Temozolomide
MGMT: O6-methylguanine-DNA methyltransferase
GSC: Glioma stem cells
AMPK: Adenosine Monophosphate-Activated Protein Kinase
MET: Metformin
ROS: Reactive Oxygen Species
HIF: Hypoxia Inducible Factor
