# Supplementary material for: Metformin and temozolomide, a synergic option to overcome resistance in glioblastoma multiforme models
Source: Oncotarget. 2017 Dec 6;8(68):113090–104. doi: 10.18632/oncotarget.23028 (PMC5762574; doi:10.18632/oncotarget.23028)
Supplement: Supplementary file 1 [file oncotarget-08-113090-s001.pdf]

Metformin and temozolomide, a synergic option to overcome resistance in glioblastoma multiforme models

SUPPLEMENTARY MATERIALS

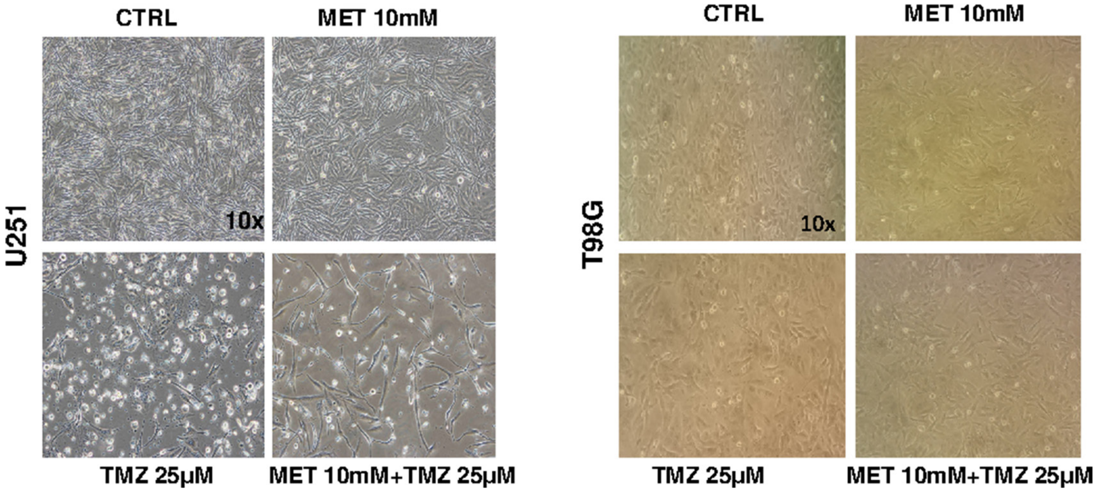

Supplementary Figure 1: Captured (10 ×) for the evaluation of the drug impact on cell viability in U251 (left panel) and T98G (right panel) after 48 h of treatment with TMZ 25 µM and/or of MET (Leyca).

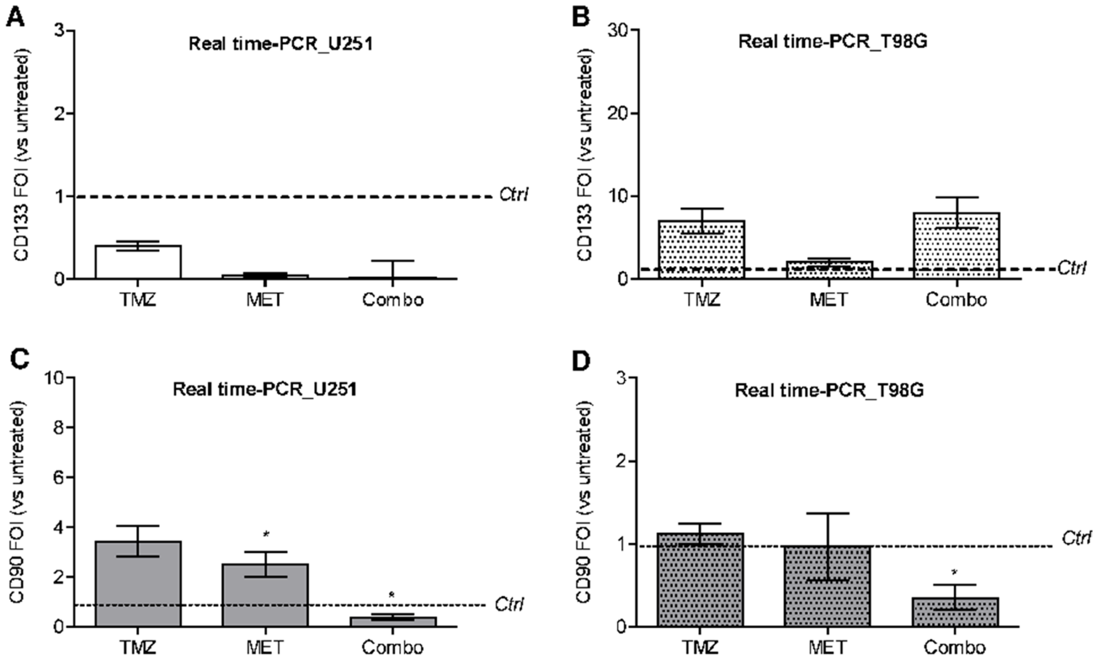

Supplementary Figure 2: **(A–D)** Real time-PCR for CD133 and CD90 genes 48 h after treatment with TMZ and/or MET. Data were normalized for β-actin and ΔΔct expressed as of Fold of Induction (FOI). $p < 0.05$ vs control sample (untreated cells).

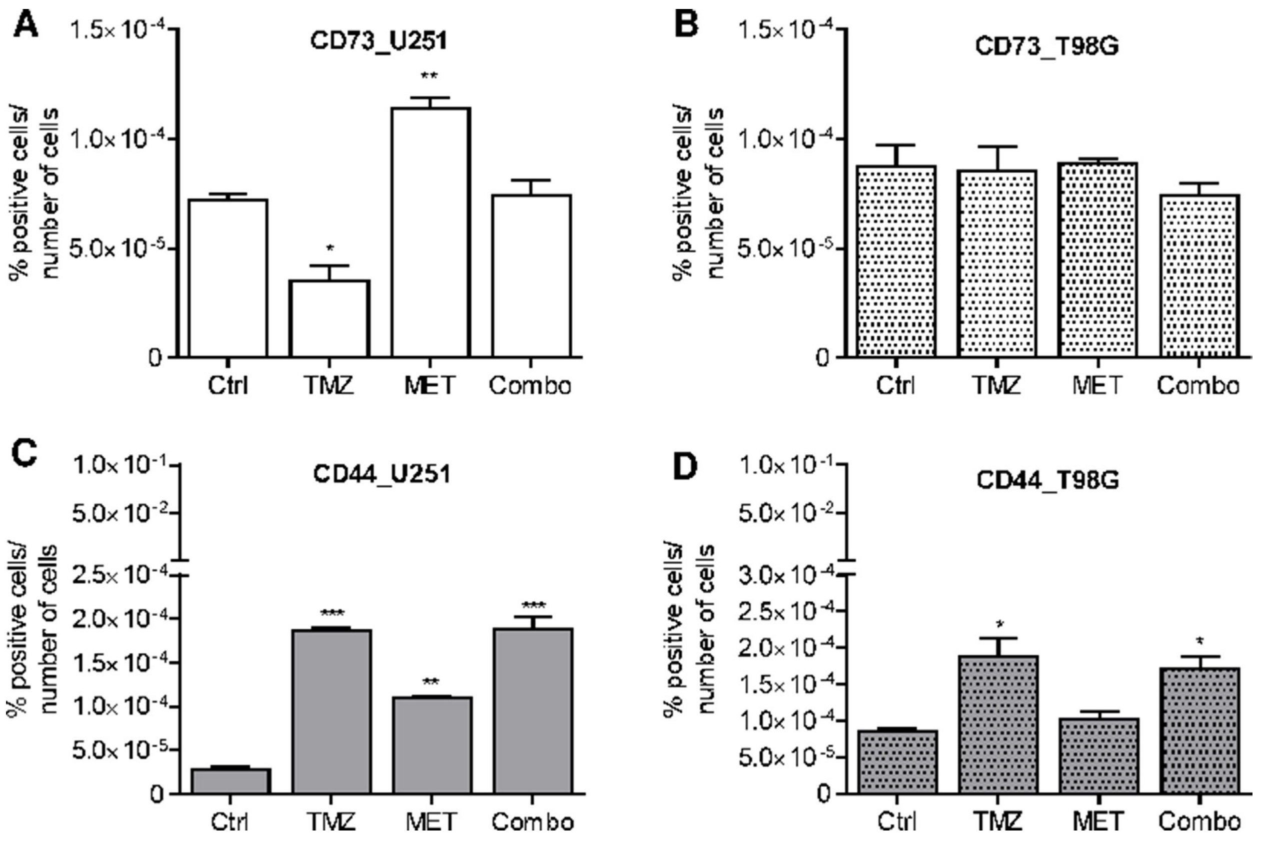

Supplementary Figure 3: FACS analysis for CD73 and CD44 markers in U251 (**A** and **C**) and T98G cells (**B** and **D**) after 48 h of treatment with TMZ 25 µM and/or of MET. Data were expressed as percentage of positive cells normalized to the number of cells. Data are shown are mean ± standard deviation. $^{*}p < 0,05$; $^{**}p < 0.01$; $^{***}p < 0.001$ vs control sample (untreated cells).

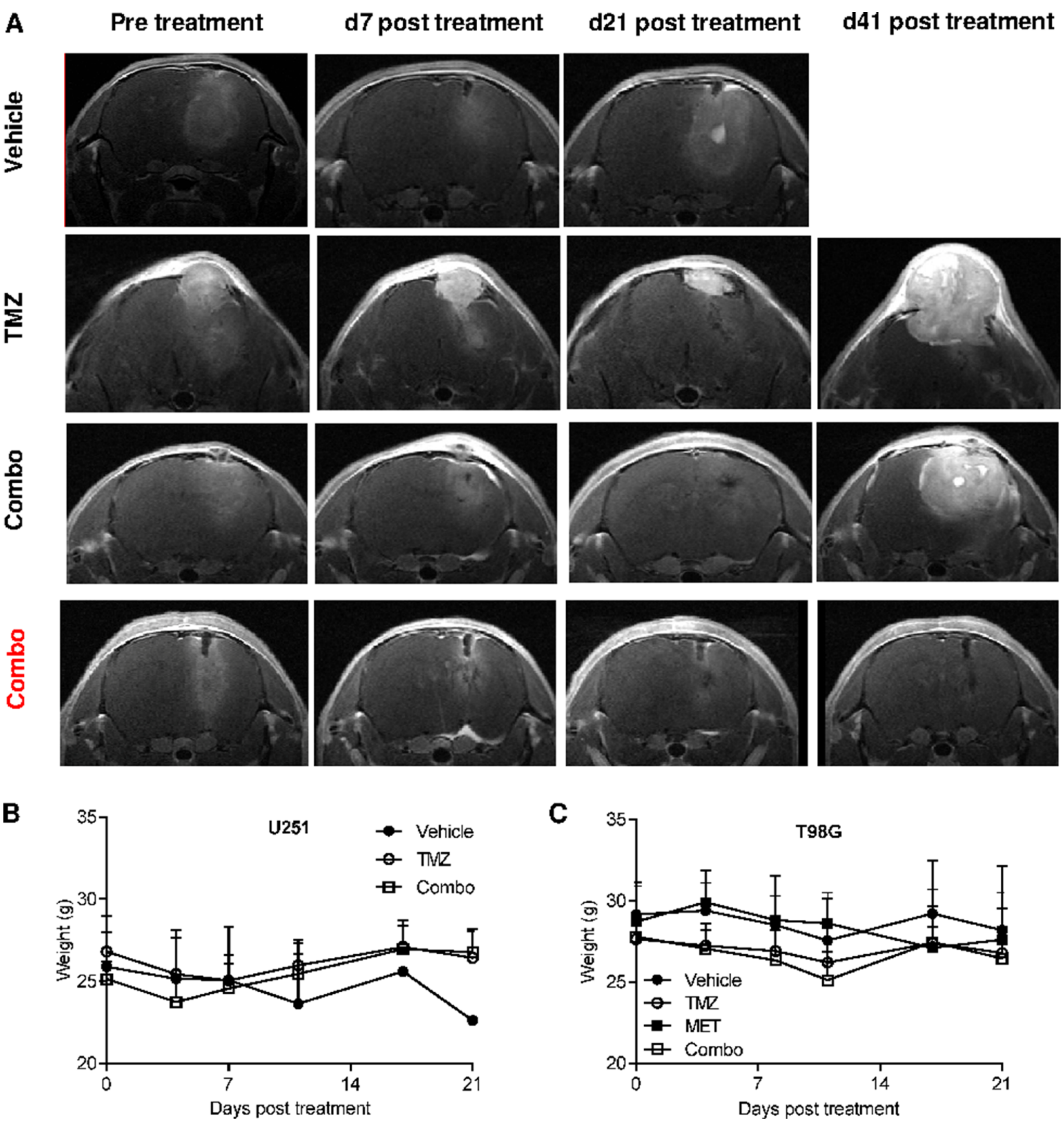

Supplementary Figure 4: Representative coronal contrast-enhanced T1 MRI images of U251 mouse model treated with vehicle, TMZ or COMBO therapy, at different time points from cell injection (**A**). Graphs of U251 and T98G animal weigh (**B**). Data are expressed as mean ± standard deviation.

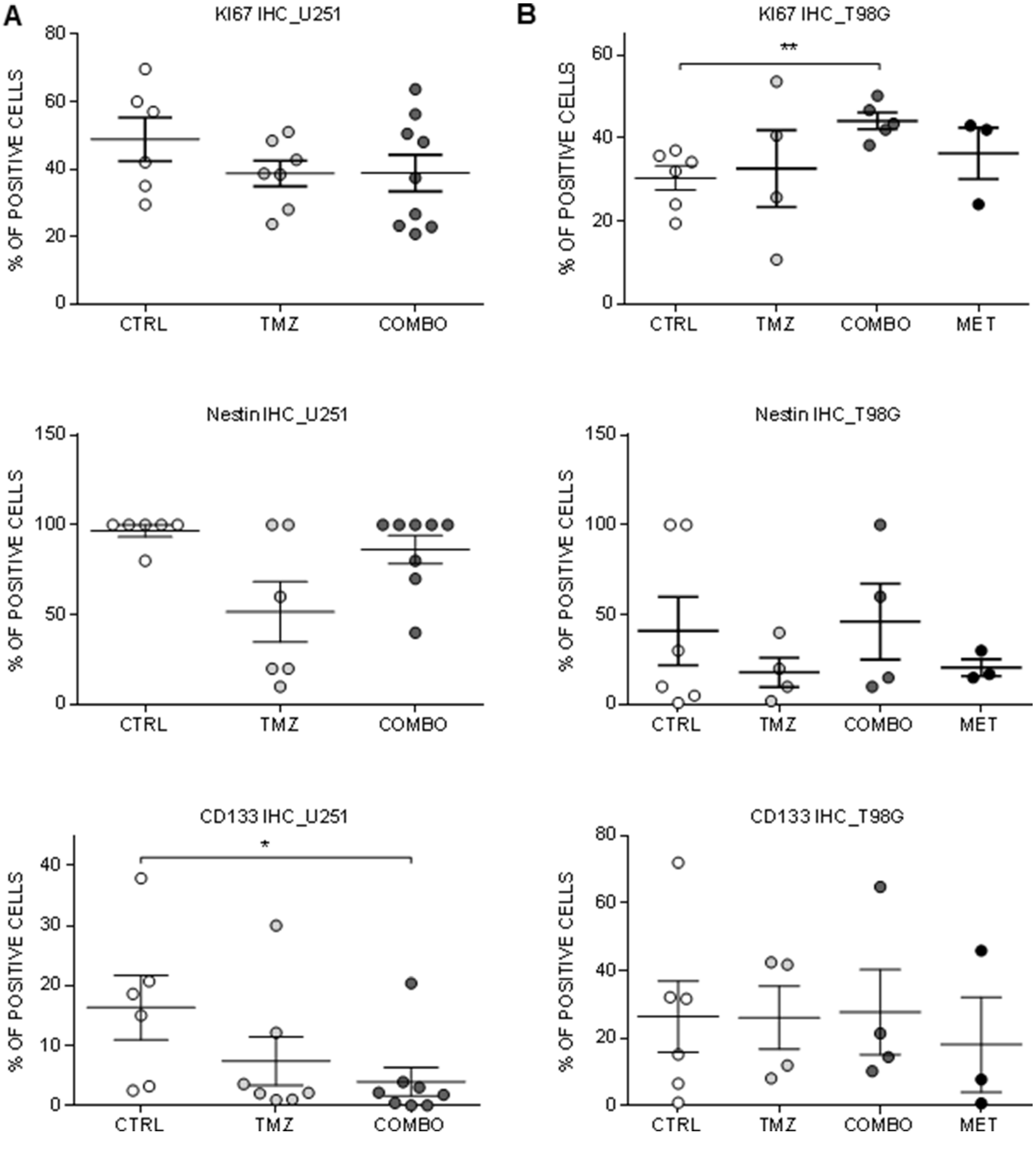

Supplementary Figure 5: Graphs show immunohistochemistry quantification of Ki67, Nestin and CD133 staining in U251 (**A**) and T98G (**B**) glioma. Bars indicate mean ± SEM. $^{*}p$ = 0,028; $^{**}p$ = 0,0043 by Mann-Whitney *U* test.

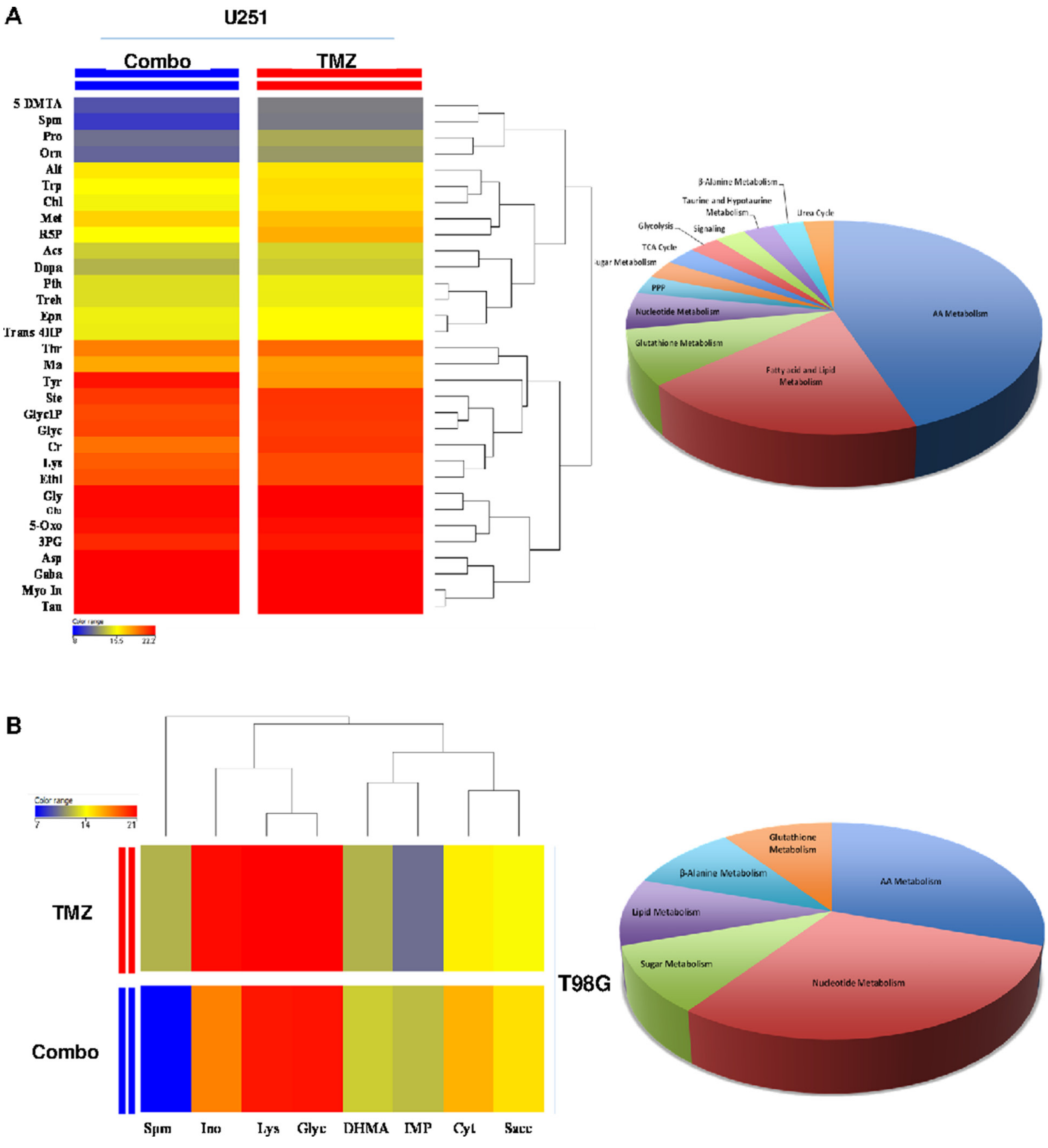

Supplementary Figure 6: (**A**) Metabolic profiling comparison of in vivo post mortem U251 samples treated with TMZ and TMZ plus Met by t-test statistical analysis. (**B**) Comparison of ex vivo T98G samples treated with TMZ and TMZ plus Met by t-test statistical analysis. *t*-Test statistical analysis was performed using Mass Profiler Professional (MPP) software. The dendrogram was produced by applying a hierarchical clustering algorithm. The color range legends was automatically generated by MPP, considering the minimum and maximum values of most compounds identified to highlight the best differences between samples through the most suitable color scale.
